# Letter to the editor in response to ‘Molecular detection of rabies virus strain with N-gene that clustered with China lineage 2 co-circulating with Africa lineages in Monrovia, Liberia: first reported case in Africa’

**DOI:** 10.1017/S0950268819001985

**Published:** 2019-12-11

**Authors:** Lifeng Zhao, Teng Chen, Faming Miao, Junfeng Li, Ying Wang, Haijun Du, Jinghui Zhao

**Affiliations:** 1 College of Animal Veterinary Medicine, Jilin Agricultural Science and Technology University, Jilin 132101, China; 2 Key Laboratory of Jilin Province for Zoonoses Prevention and Control, Laboratory of Epidemiology, Institute of Military Veterinary Medicine, Academy of Military Medical Sciences, Changchun 130122, China; 3 Jilin Agricultural University, Changchun 130118, China; 4 Changchun Sci-Tech University, Changchun 130600, China

To the Editor

With great interest, we read the article by Olarinmoye *et al*. titled ‘Molecular detection of rabies virus strain with N-gene that clustered with China lineage 2 co-circulating with Africa lineages in Monrovia, Liberia: first reported case in Africa’ [1]. Two technical issues should be considered.

First, the full-length of the nucleoprotein (N) and the glycoprotein (G) gene should get sequenced to perform sequence analysis on the proteins. The replacement of the amino acid in comparison with other rabies viruses should be further investigated.

**Fig. 1.**
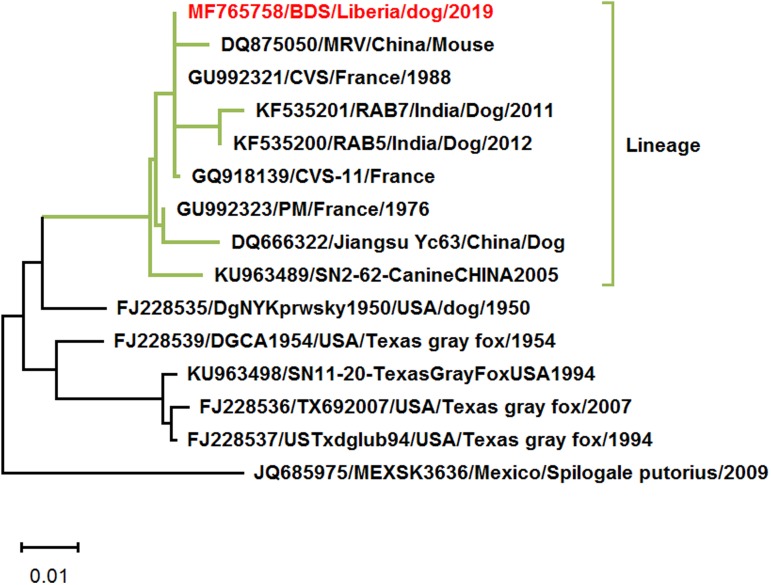
Maximum likelihood phylogenetic tree on the partial N gene of rabies viruses. This analysis involved 15 nucleotide sequences. Evolutionary analyses were conducted in MEGA X.

Second, the result of phylogenetic analysis, ‘the rabies virus (RABV) strain (MF765758) detected clustered with China lineage 2 RABVs of dogs (99% homology to KU963489 and DQ666322)’, is not comprehensive and need further analysis. Based on the partial N gene sequence (MF765758), we Blast it in the NCBI GenBank (https://blast.ncbi.nlm.nih.gov/Blast.cgi). The partial of 15 nucleoprotein (N, 554-nt) genes retrieved from the NCBI GenBank database. Multiple alignments of sequences were performed using CLUSTAL X 2.1 [2], and the aligned sequences were used to infer the phylogenetic tree by maximum likelihood (ML) methods using MEGA X [3]. Apart from the two RABV strains of China lineage 2 (KU963489 and DQ666322), the France RABV strain, CVS (GU992321), the India RABV strains, RAB5 and RAB7 (KF535200 and KF535201), had very close resemblance (99% homology) with Monrovia RABV sequence MH765758. The phylogenetic analysis revealed them to be the same lineage (Fig. 1). China RABV strains KU963489 (or SN2-62-CanineCHINA2005) and DQ666322 (or Jiangsu_Yc63) were not pandemic strains in China [4]. The phylogenetic analysis would be more comprehensive and accurate if RABV strains from India and France were added.

It is worth noting that the relationship between Liberia strain (MH765758), India strains (KF535200 and KF535201), China strains (KU963489, DQ666322 and DQ875050) and France RABV strains (GU992321 and GQ918139) should be further investigated.
